# Assessment of Spectral Doppler for an Array-Based Preclinical Ultrasound Scanner Using a Rotating Phantom

**DOI:** 10.1016/j.ultrasmedbio.2015.04.006

**Published:** 2015-08

**Authors:** David A. Kenwright, Tom Anderson, Carmel M. Moran, Peter R. Hoskins

**Affiliations:** Centre for Cardiovascular Science, University of Edinburgh, Edinburgh, United Kingdom

**Keywords:** Doppler ultrasound, Blood velocity, High-frequency ultrasound, Preclinical ultrasound, Doppler phantom

## Abstract

Velocity measurement errors were investigated for an array-based preclinical ultrasound scanner (Vevo 2100, FUJIFILM VisualSonics, Toronto, ON, Canada). Using a small-size rotating phantom made from a tissue-mimicking material, errors in pulse-wave Doppler maximum velocity measurements were observed. The extent of these errors was dependent on the Doppler angle, gate length, gate depth, gate horizontal placement and phantom velocity. Errors were observed to be up to 172% at high beam–target angles. It was found that small gate lengths resulted in larger velocity errors than large gate lengths, a phenomenon that has not previously been reported (*e.g.,* for a beam–target angle of 0°, the error was 27.8% with a 0.2-mm gate length and 5.4% with a 0.98-mm gate length). The error in the velocity measurement with sample volume depth changed depending on the operating frequency of the probe. Some edge effects were observed in the horizontal placement of the sample volume, indicating a change in the array aperture size. The error in the velocity measurements increased with increased phantom velocity, from 22% at 2.4 cm/s to 30% at 26.6 cm/s. To minimise the impact of these errors, an angle-dependent correction factor was derived based on a simple ray model of geometric spectral broadening. Use of this angle-dependent correction factor reduces the maximum velocity measurement errors to <25% in all instances, significantly improving the current estimation of maximum velocity from pulse-wave Doppler ultrasound.

## Introduction

**Fig. 1:**
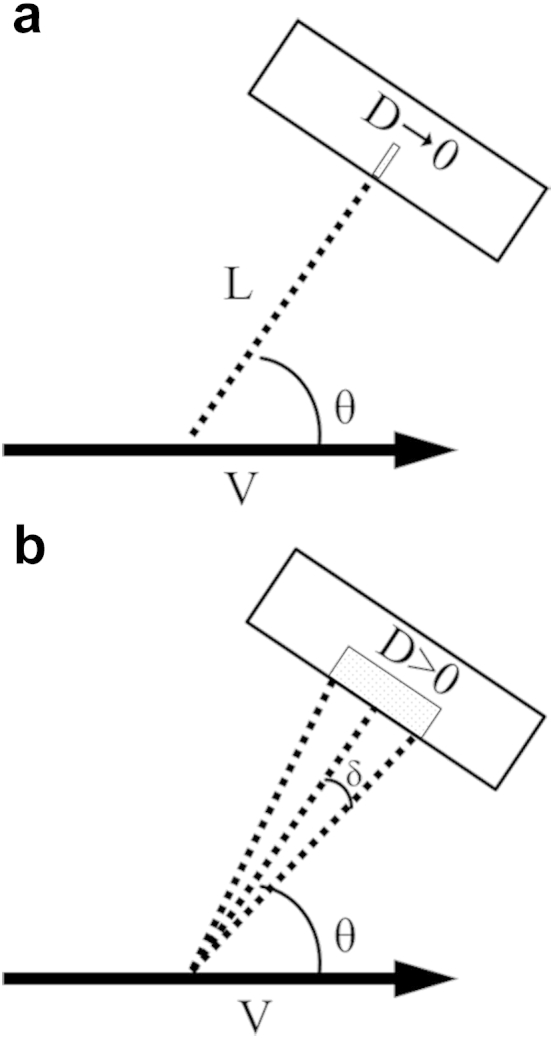
(a) The Doppler equation, used to calculate the velocity *V* of a moving target from the Doppler shift in the transmitted and received ultrasound signal, assumes the transducer is at a distance *L* from the target at an angle to the ultrasound beam θ and does not take into account the size of the aperture *D*. (b) In reality, the aperture has a finite width (*D* > 0), and therefore, there are a range of angles (θ − δ to θ + δ) that the beam subtends, causing a spread in the received Doppler shift from a target moving with constant velocity.

Doppler ultrasound provides a means to measure blood velocity and is used in both research and clinical investigations to quantify the extent and effect of arterial disease. Applications include determination of the degree of stenosis for determining stroke risk (Grant et al. 2003); the downstream resistance to flow to assess renal haemodynamics (Chen et al. 1993); volumetric blood flow, also requiring a measurement of arterial diameter (Alvarez et al. 1993); and wall shear stress as a potential indicator of atherogenic risk (Blake et al., 2008, Yang et al., 2013a). Velocity measurements are typically derived from either the mean or maximum frequency of the Doppler spectrum. The mean frequency is very sensitive to the placement of the sample volume within the flow field, and movement artefacts such as transducer motion or vessel displacements between cardiac cycles can cause the sample volume to move relative to the vessel. In contrast, the maximum frequency of the Doppler spectrum is less likely to change with sample volume placement, and thus, measurements of blood velocity often use the maximum Doppler shift. However, ultrasound systems are susceptible to high measurement errors in maximum blood velocity. For clinical systems, this error has been found to be typically in the range 0%–60%; however, this can increase to >100% when the Doppler angle approaches 80°–90° (Hoskins, 1996, Hoskins, 1999). Although the misalignment of the ultrasound beam within the target vessel is corrected for with the angle cursor *via* the Doppler equation, this assumes that the ultrasound beam is received at a single, narrow point on the array (Fig. 1a), when in reality the aperture of a transducer is of a finite width, causing the target velocity vector to subtend a range of angles (Fig. 1b) that are not accounted for (Newhouse et al. 1980). This phenomenon is known as *geometric spectral broadening* and has been reported to be the main source of error in maximum velocity estimation (Hoskins, 1999, Hoskins et al., 1999).

High-frequency ultrasound is a powerful tool in small animal anatomic and functional *in vivo* imaging because it has high resolution, occurs in real time, is free from ionising radiation and is relatively inexpensive. It is increasingly used as an imaging modality in preclinical investigations, where preclinical relates to models of human disease and often uses small animals such as mice, rats and zebrafish (Goertz et al., 2002, Goessling et al., 2007, Heiss et al., 2008). The first commercially available preclinical imaging system utilised mechanically swept single-element transducers (Foster et al. 2002). A recent study found that for this system, the maximum velocity from spectral Doppler was overestimated by up to 158%, with good agreement with errors predicted from geometric spectral broadening at high beam–target angles (Yang et al. 2013b). As each single-element transducer has a fixed focal depth, the range of useful angles that can be obtained for spectral Doppler measurements is limited. An array-based preclinical system has been developed (Foster et al. 2009). Multiple focal depths can be achieved with electronic focus of the array elements, such that the beam characteristics can be optimised for Doppler measurements over a variety of depths.

Unlike the clinical situation, to date there is limited information on velocity errors in high-frequency ultrasound applications. In this article, we use a small-size rotating phantom to investigate, for the first time, the velocity errors in an array-based preclinical ultrasound scanner, which is predicted to suffer from the same limitations as lower-frequency clinical systems.

## Methods

### Ultrasound scanner

Ultrasound scanning was performed using a Vevo 2100 high-frequency ultrasound scanner (FUJIFILM VisualSonics, Toronto, ON, Canada) with a MS-550 D linear-array probe with a central frequency of 40 MHz (broadband frequency: 22–55 MHz). The probe could be set to operate at 32 MHz (default value) or 40 MHz.

### Rotating phantom

**Fig. 2:**
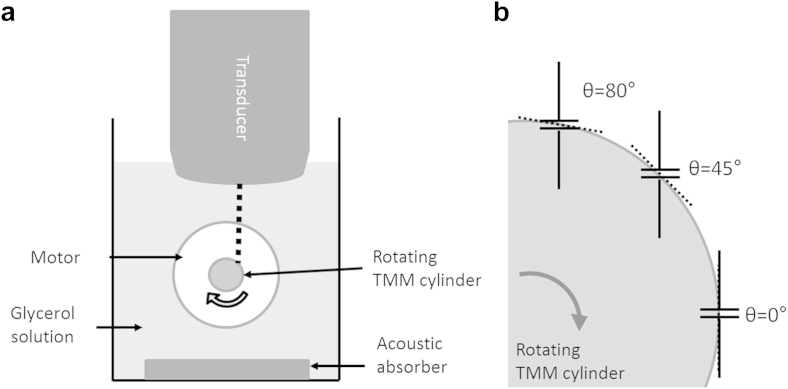
(a) Experimental setup. The *dashed line* represents the ultrasonic beam. (b) The sample gate is acquiring Doppler measurements at three different angles on the surface of the rotating phantom. The *dashed lines* represent the angle cursor aligned tangentially to the surface of the rotating TMM cylinder. TMM = tissue-mimicking material.

A miniature rotating phantom composed of tissue-mimicking material (TMM) was created as described by Yang et al. (2013b). The TMM was developed for use with clinical ultrasound systems (Teirlinck et al. 1998) and was recently characterised at high acoustic frequencies (Rajagopal et al., 2015, Sun et al., 2012). Briefly, a cylinder of TMM was set in a mould (inner diameter = 6 mm) on a nylon drive wheel, supported by projecting loop of copper wire. Once the TMM had set, the mould was removed. The drive wheel with the TMM was attached to the drive shaft of the motor of a modified string phantom (BBS Medical Electronics, Hägersten, Sweden). The motor was controllable to provide a constant rotational velocity. The TMM provided ultrasound backscatter such that a Doppler signal could be obtained from the outer edge. The phantom was submerged in a tank filled with 9% glycerol solution by volume, which had an acoustic velocity of 1540 m/s at 20°C. An acoustic absorber pad was placed underneath the phantom to reduce ultrasound reflections. The experimental setup is illustrated in Figure 2a.

### Experimental measurements

Pulse-wave (PW) spectral Doppler measurements of velocity were carried out while varying five different parameters: measurement angle; sample gate length; measurement depth (distance from transducer to sample gate); lateral position of the sample gate along the face of the array; and velocity of the rotating phantom. In each case the true linear velocity (*V*_true_) was obtained by measuring the period of rotation (*T*) from a spike in the Doppler trace caused by an indentation in the surface of the TMM, such that

(1) $$V_{\text{true}}=\pi d/T$$

where *d* is the diameter of the TMM cylinder (6 mm). The percentage error in the measured velocity (*V*_err_) from Doppler ultrasound (*V*_Doppler_) was therefore

(2) $${\text{V}}_{\text{err}}=\frac{\left(V_{\text{Doppler }}-\;V_{\text{true}}\right)}{V_{\text{true}}}\times 100$$

Velocity measurements from PW Doppler were obtained by using the maximum Doppler frequency trace averaged over 5 s using the on-board analysis software on the VisualSonics scanner. The maximum frequency, which originates from the outer edge of the rotating phantom from which the velocity was estimated, was used. Figure 2b is a schematic indicating how Doppler measurements were obtained from the surface of the TMM.

### Angle dependency

To assess the angle dependence of velocity measurements, the Doppler gate was located at a fixed depth of 6 mm in the centre of the display (*i.e.,* with the aperture originating from the centre of the array). The gate length was set to the minimum (0.12 mm at 32 MHz, 0.11 mm at 40 MHz). Measurements were taken at multiple positions along the outer edge of the rotating phantom, with the probe being moved vertically and laterally to maintain the same depth and lateral position of the Doppler gate. At each position, the angle cursor was adjusted to be tangential to the surface of the TMM. Thus, velocity measurements could be taken on the surface of the TMM at angles between 0° and 80° with a step size of 5°, as limited by the on-board software. Four independent measurements were taken at each angle. Measurements were repeated with the probe set to operate at 32 and 40 MHz.

### Sample volume dependency

To determine sample volume dependency, the above measurements were repeated with the probe at the default frequency (32 MHz) and the Doppler gate set to four different lengths: 0.12 mm (minimum gate length), 0.22 mm, 0.51 mm and 0.94 mm (maximum gate length). Four independent measurements were taken at each gate length.

### Depth dependency

To assess the depth dependence of velocity measurements, measurements were taken with the tangential surface of the TMM at 45° to the ultrasound beam (with the Doppler angle also fixed at 45°), chosen as being the typical obtainable angle during *in vivo* Doppler measurements. The probe was then moved vertically at 1-mm intervals, and the Doppler gate manually repositioned to the outer edge of the moving phantom. Measurements were made with the aperture originating from the centre of the array. Four independent measurements were taken at each depth, with the probe set to operate at both 32 and 40 MHz.

### Lateral aperture position dependency

For lateral position dependency, the sample gate was placed at multiple horizontal positions within the imaging window (and therefore along the face of the array) while keeping the depth fixed at 6 mm, the default depth and also typical of measurement depth used in preclinical practice. The lateral position was determined off-line using the pixel coordinates of the centre of the sample volume from a TIFF screenshot of the measurements. Measurements were binned into 2-mm intervals at 32 MHz and 1.4-mm intervals at 40 MHz. All measurements were taken at a fixed angle of 45°. Measurements were taken left-to-right twice and right-to-left twice.

### Velocity dependency

Finally, the velocity of the rotating phantom was varied between 2 and 27 cm/s while measurements were taken at a fixed depth of 6 mm and a fixed angle of 0°, and the velocity error was calculated.

### Theoretical error from geometric spectral broadening

In estimating blood velocity, the Doppler equation was used in the form

(3) $$V=\frac{cf_{\text{d}}}{2f_{\text{t}}\;\cos\;\theta^{'}}$$

where *c* is the estimated speed of sound in tissue (generally 1540 m/s), *f*_d_ is the detected Doppler shift frequency, *f*_t_ is the transmitted frequency and θ is the angle between the path of the ultrasound beam and the direction of the blood flow. The angle θ assumes that the ultrasound beam is received at a single, narrow point, when in reality a range of angles (θ − δ to θ + δ) are subtended across the width of the ultrasonic aperture.

Using a simple model of geometric spectral broadening (Hoskins 1999), we can take into account the largest angle subtended by the ultrasound beam to the aperture and hence we can estimate that the error in the maximum velocity measurement (*V*_err_) from PW Doppler can be given as

(4) $$V_{\text{err}}=\left(D/2L\right)\tan\;\theta$$

where *D* is the aperture width of the transducer, *L* is the measurement depth and θ is the beam–target angle. For the full derivation, see Yang et al. (2013b). The aperture width can be estimated from the *f*-number (or focal ratio) *N*, where *N* = *L*/*D*. For the Vevo 2100, the software attempts to maintain a constant *f*-number of 2 by adjusting the aperture width with depth (communications with the manufacturer). On this basis, we test a correction for the effects of geometric spectral broadening such that the corrected velocity *V*_corr_ is given by

(5) $$V_{\text{corr}}=V_{\text{measured}}\times \text{CF},$$

where *V*_measured_ is the measured velocity and CF is a correction factor:

(6) $$\text{CF}=\frac{1}{\left(D/2L\right)\tan\;\theta +1}$$

## Results

**Fig. 3:**
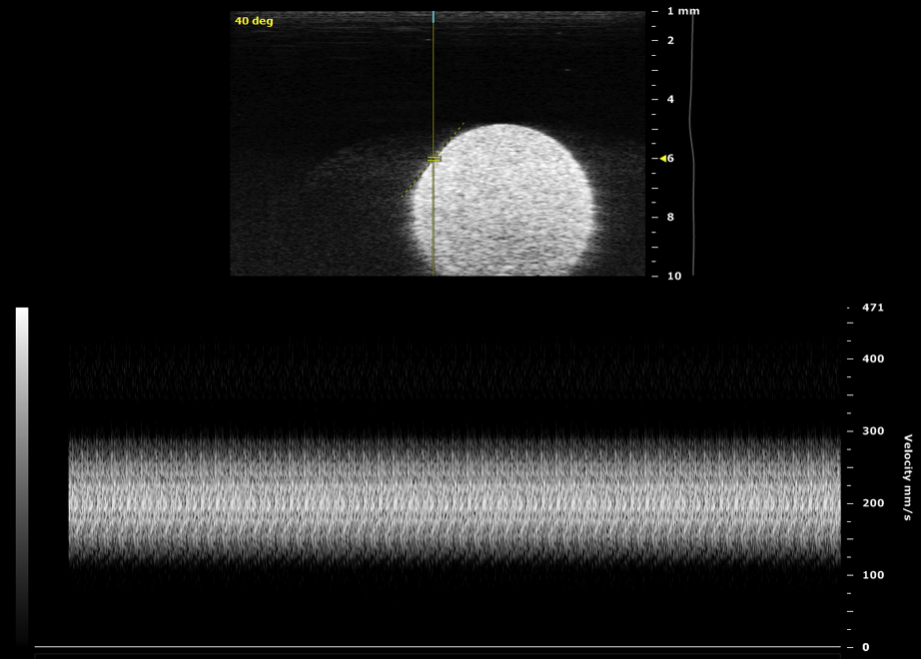
B-Mode and pulse-wave Doppler measurements of the rotating phantom at 40° and a depth of 6 mm, with the probe set to 32 MHz and the sample volume size 0.12 mm (*i.e.*, minimum for 32 MHz).

In Figure 3 are example B-mode and Doppler spectrum measurements of the rotating phantom.

**Fig. 4:**
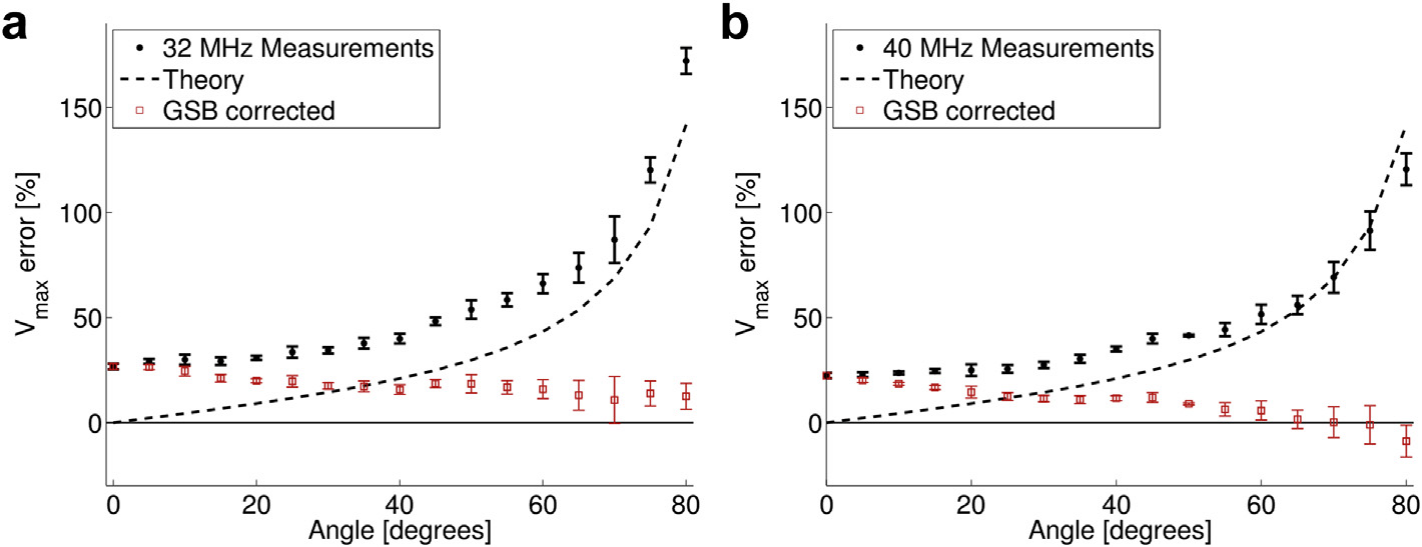
Error in the maximum velocity measurement as a function of the beam–target angle for transducers set to (a) 32 MHz and (b) 40 MHz (*black circles*). The theoretical expected error based on a zero-beam-width model of GSB is indicated by the *dashed lines*. The error after the application of a correction factor based on geometric spectral broadening reduces the measured error to <22% (*red squares*). GSB = geometric spectral broadening.

**Fig. 5:**
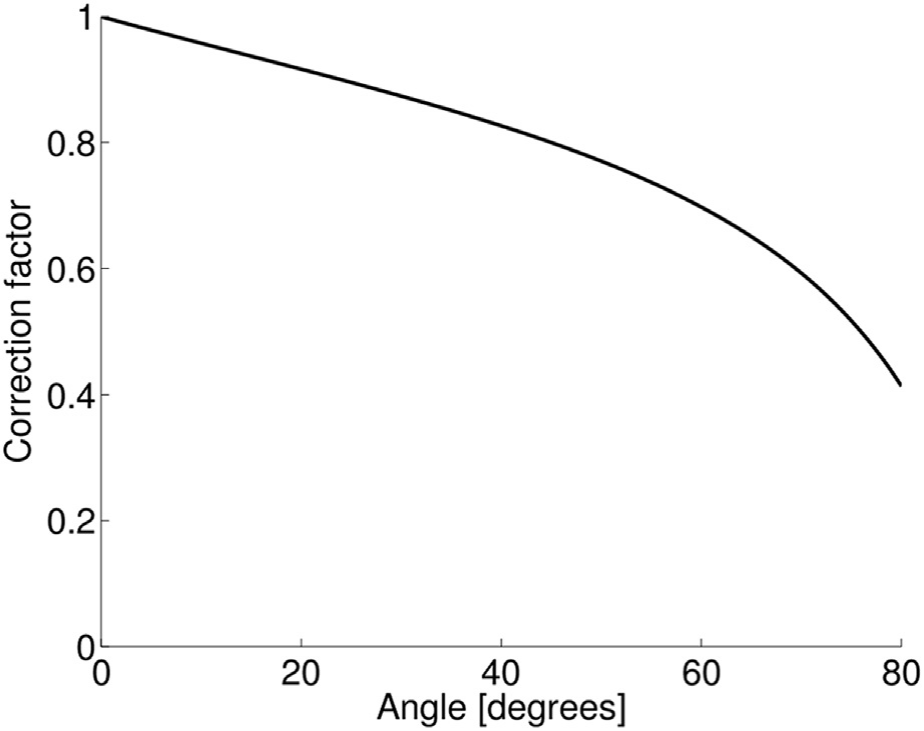
Correction factor derived from the theoretical effect of geometric spectral broadening.

Figure 4 illustrates the estimated maximum velocity error from the MS-550 D as a function of measurement angle for both operating frequencies at the minimum sample volumes. The measured values were compared with the theoretical error from geometric spectral broadening. A correction factor as a function of beam–target angle was derived from this theoretical error (Fig. 5). The measured velocity was then multiplied by the correction factor to give a corrected velocity (Fig. 4, *red squares*). For both operating frequencies of the probe, the measured error increased with angle up to 172%. With corrections for geometric spectral broadening, the error was improved for all angles >0°. When the probe was set to 32 MHz and with corrections for geometric spectral broadening, the error was 18 ± 5%. When the probe was set to 40 MHz, the measured error was 10 ± 8%, and agrees more closely with the theory at higher angles.

**Fig. 6:**
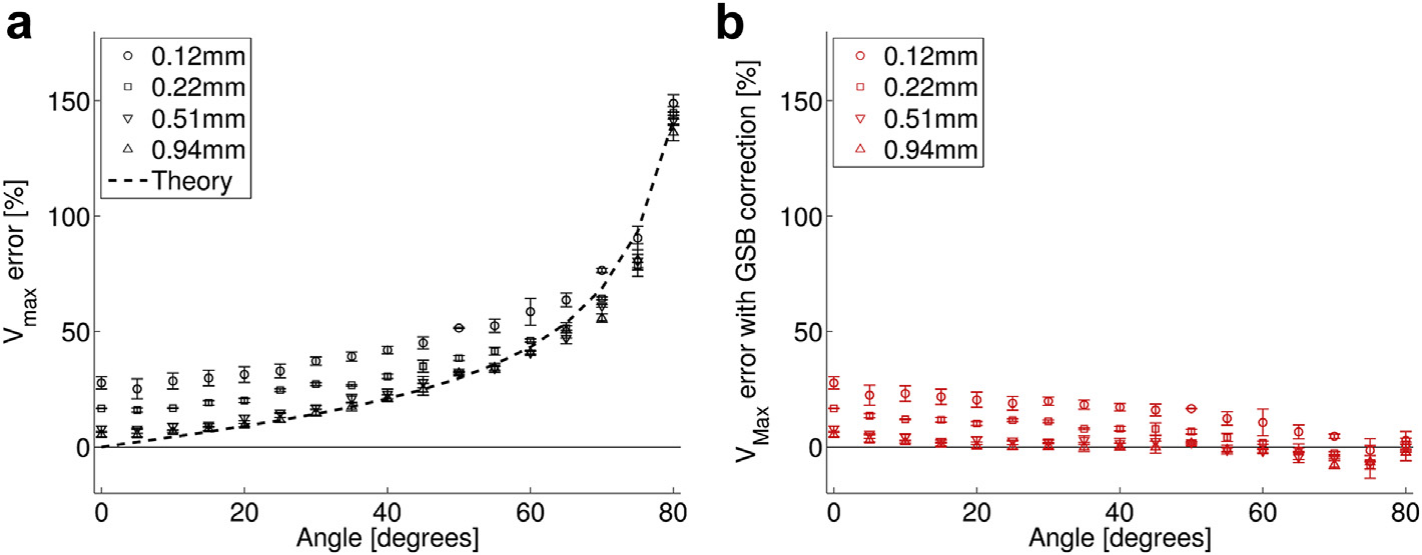
Error in maximum velocity measurement as a function of beam–target angle with (a) changing sample volume size and (b) after correction for theoretical error from GSB. The measured error more closely matches the theoretical error from geometric spectral broadening at larger sample volume sizes, with improved agreement at larger angles. Probe set to 32 MHz. GSB = geometric spectral broadening.

**Fig. 7:**
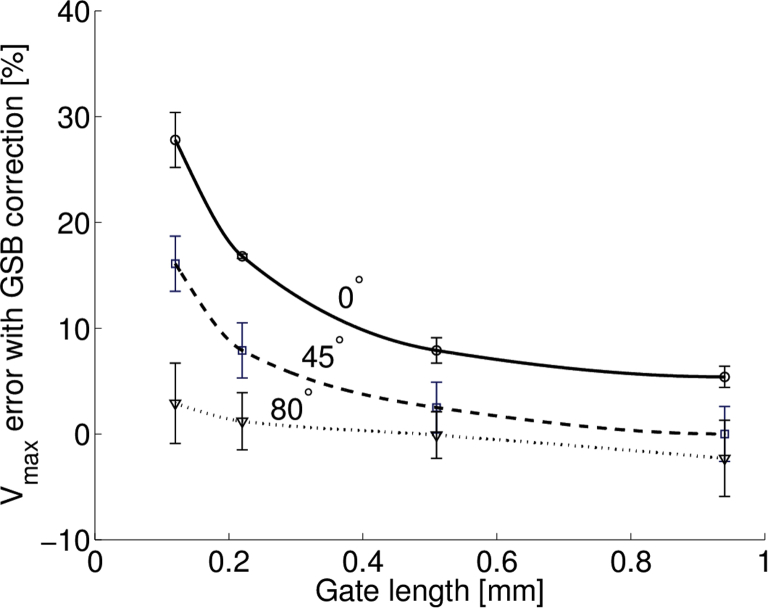
Error in maximum velocity measurement as a function of sample volume. Probe set to 32 MHz.

Figure 6 illustrates how the error in maximum velocity estimation changed with angle for four different gate lengths before (Fig. 6a) and after (Fig. 6b) correcting for geometric spectral broadening with the correction factor. The greatest difference appeared at lower angles, and as the gate length was increased, the agreement with theoretical error improved. For example, with a beam–target angle of 0°, the error in the maximum velocity measurement was 27.8% when measured with a 0.2-mm gate length and 5.4% with a 0.98-mm gate length. Figure 7 illustrates that the difference in velocity error decreased with increasing gate length.

**Fig. 8:**
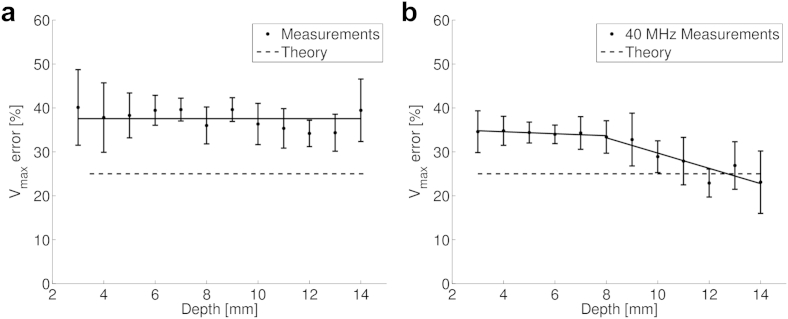
Error in maximum velocity measurement as a function of gate depth at (a) 32 MHz and (b) 40 MHz. As all measurements are at a fixed angle (45°), the theoretical error from geometric spectral broadening is constant. The *solid lines* are lines of best fit. For the 40-MHz probe, two lines of best fit are shown to illustrate the changing trend at increased depth.

Figure 8 illustrates velocity error as a function of the gate depth at both operating frequencies for measurements at a Doppler angle of 45°. There was little change in the error when the probe was operating at 32 MHz, but at 40 MHz, there was a decrease in error at depths >9 mm.

**Fig. 9:**
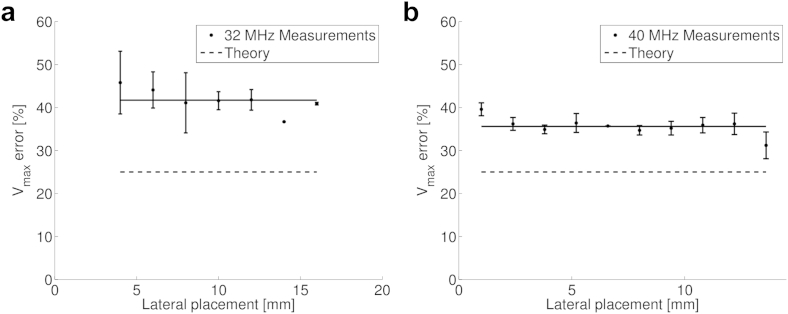
Error in maximum velocity measurement as a function of the lateral position of the gate along the face of the transducer array at 32 MHz (left) and 40 MHz (right). The *solid lines* indicate the mean velocity error.

Figure 9 illustrates velocity error as a function of horizontal sample gate position along the array at 32 MHz (Fig. 9a) and 40 MHz (Fig. 9b) for measurements at a depth of 6 mm and a Doppler angle of 45°. At 40 MHz there was a change in velocity error at the extreme edges of the position: an increase in the error on the left side, and a decrease in the error on the right side.

**Fig. 10:**
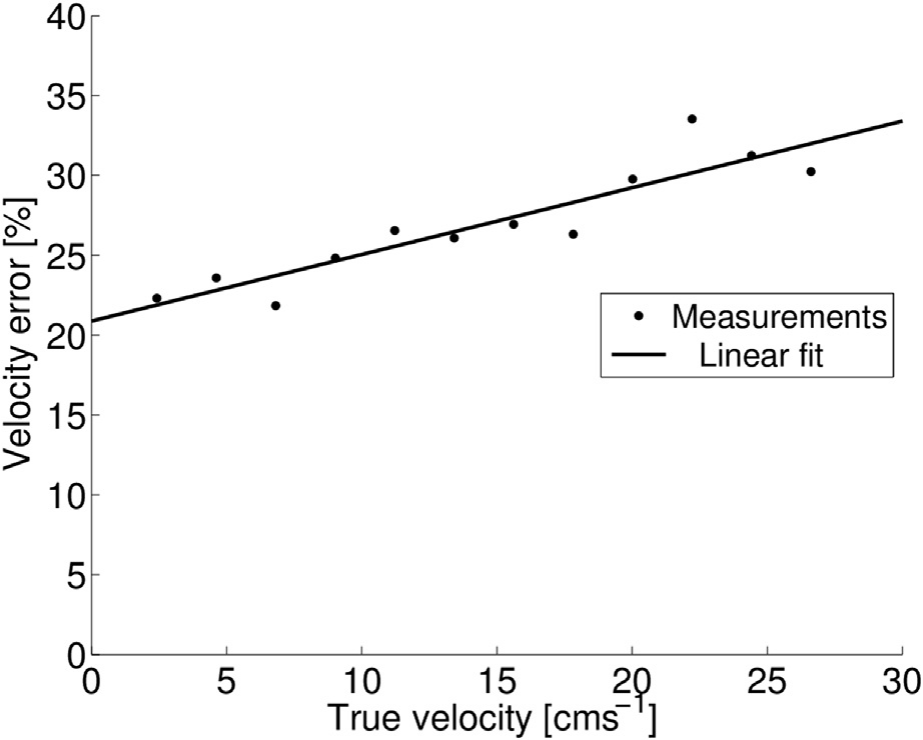
Error in maximum velocity measurement as a function of the true velocity of the rotating phantom. The *solid line* is a linear fit to the data. Probe set to 32 MHz.

Figure 10 illustrates velocity error for a set depth (6 mm), angle (0°), sample volume (0.12 mm) and lateral position (centre), as a function of the true velocity of the rotating phantom. The error ranged from 22% at 2.4 cm/s to 30% at 26.6 cm/s. The velocity error increased approximately linearly (*R*^2^ = 0.83) as the true velocity of the phantom increased.

## Discussion

In this study we found that PW Doppler measurements of velocity on an array-based preclinical ultrasound system can be overestimated by up to 172%, with higher errors at higher angles. We compared the magnitude of this error with the error predicted from a simple ray model of geometric spectral broadening, which takes into account the usually neglected width of the aperture of the transducer during maximum velocity estimation. The variable size of the aperture width in array-based transducers is generally seen as beneficial as it can provide multiple focal depths and optimal positioning for different measurement locations, but also introduces an additional source of error. The assumption was made that the error in the maximum velocity measurement would be similar to that seen in clinical systems (Hoskins 1999) and the single-element preclinical system (Yang et al. 2013b). Previously, the difference between finite-beam-width and zero-beam-width models was found to be small, between 0.5% and 2%, for a single-element high-frequency scanner (Yang et al. 2013b), therefore, a zero beam width was assumed at the focus. The observed error is consistent with what has previously been observed in clinical systems and an earlier single-element preclinical system.

We have noted a strong dependency of estimated maximum velocity on Doppler gate length, which to our knowledge has not been previously reported for PW spectral Doppler measurements. The error is reduced when the gate length is increased and more closely matches errors resulting from geometric spectral broadening. This effect has not been previously reported for either clinical or preclinical ultrasound systems. One possible explanation is that it is due to the frequency estimation at low sample volumes, which will be broadened because of the short length of the observation of the pulse. This would increase the maximum velocity estimation and, therefore, account for the greater overestimation. An investigation into this phenomenon could be undertaken through simulation of the Doppler measurement process (*e.g.*, Jensen, 1996, Van Canneyt et al., 2013), but is beyond the scope of this article.

Previous studies have reported that the variation in velocity error with Doppler gate depth in clinical systems is due to a change in the *f*-number (Hoskins 1999). For the preclinical system of this study, there was no variation in error with gate depth at 30 MHz, suggesting that the *f*-number remains constant (*i.e.,* the Doppler aperture is increased in proportion with the gate depth). At 40 MHz, the velocity error is initially constant with increasing depth up to 9 mm, but the error decreases for greater depths. This suggests that the f-number is initially maintained constant, but decreases for depths >9 mm. The most likely explanation is that the Doppler aperture size increases to a depth of 9 mm and is maintained constant beyond 9 mm. Similarly, we have observed small changes in the velocity estimation with horizontal placement of the sample gate when the probe is operating at 40 MHz. This may indicate that some change in the aperture size is effected by the on-board software as the extreme edges of the measurement window are reached, as previously observed in clinical ultrasound systems (Hoskins et al. 1999). Future work that measures the aperture width of the transducer for different measurement positions would provide useful information, but is outside the scope of this article.

We derived a correction factor based solely on geometric spectral broadening, using knowledge of the measurement depth and beam–target angle (both typically displayed by default on commercial ultrasound systems) and the aperture width, which can be estimated from the *f*-number, which has been found to remain constant for a probe operating at 32 MHz and appears to increase at large depths when operating at 40 MHz. The manufacturer confirmed that the on-board software attempts to maintain an *f*-number of 2 throughout the image depth and sets the aperture width (*i.e.,* number of active elements) accordingly, until the maximum aperture is reached, limited by the number of elements available. By application of this correction factor to the measured maximum velocity, the overestimation is reduced to <25% at all measured angles. Users of other ultrasound systems could use this method either by obtaining the *f*-number of the probe (not typically displayed to the user) or by measuring the aperture width using a needle hydrophone (*e.g.,* see Yang et al. 2013b). We can see no reason why such a correction factor could not be added to the on-board software of future ultrasound systems, eliminating the need for the end user to correct for these measurement errors. However, the failure of the correction factor to fully eliminate velocity errors also indicates that there is still a large measurement error (up to 25%) at low angles and small gate lengths, which means that geometric spectral broadening does not fully explain the errors in maximum velocity estimation.

A review of ultrasound blood velocity measurements (Hoskins 2002) lists three other sources of error in calculating velocity from the Doppler frequency shift, all of which come under the general term “intrinsic spectral broadening.” First, there is the variation is the velocity during the sample time or acceleration (Fish 1991). In this study, we have a fixed velocity; therefore this will be zero. “Velocity gradient broadening” is due to the variations in velocity within the sample volume under observation. Because of the cylindrical nature of the phantom used in this study, there will be additional velocity components from within the phantom (due to a finite beam width); however, these will be lower than the velocity of the outer edge and have no effect on estimation of the maximum velocity. “Transit time broadening” is associated with the length of time the moving target remains in the beam. Transit time broadening may have a greater effect in high-frequency ultrasound systems, and further work in modelling this effect would be of interest. However, geometric spectral broadening has been found to be the main source of error in maximum velocity estimation in clinical systems (Hoskins, 1999, Hoskins et al., 1999).

Human error already accounts for large errors in Doppler velocity measurements, arising from incorrect angle alignment, sample volume placement and gain settings (Lui et al. 2005). It is important to minimise such errors, and testing ultrasound equipment with phantoms can provide information on further velocity errors. Given the proliferation of ultrasound in preclinical imaging and the applications that benefit from PW Doppler measurements, the authors are of the view that the widespread use of the derived correction factor to improve the accuracy of blood velocity measurements would greatly benefit future preclinical studies. By correcting for geometric spectral broadening with a correction factor, users of preclinical ultrasound scanners in research can improve the accuracy of measurements of blood velocity compared with those provided directly by the on-board software.

## Conclusions

We have investigated errors in the maximum velocity measurements using pulse-wave Doppler for a first-generation commercial array-based preclinical ultrasound scanner. Errors consistent with geometric spectral broadening have been observed, similar to those errors seen in clinical ultrasound systems. The error is also highly sensitive to sample volume length, indicating that other sources of spectral broadening are involved. Maximum velocity measurement errors are between 25% and 172% depending on the measurement angle. By Correction for geometric spectral broadening alone can reduce measurement errors to <25% at all angles.
